# Scoping review of the methodology of large health surveys conducted in Spain early on in the COVID-19 pandemic

**DOI:** 10.3389/fpubh.2023.1217519

**Published:** 2023-08-02

**Authors:** Carmen Sánchez-Cantalejo Garrido, Daniela Yucumá Conde, María del Mar Rueda, Antonio Olry-de-Labry-Lima, Eva Martín-Ruiz, Camila Higueras-Callejón, Andrés Cabrera-León

**Affiliations:** ^1^Department of Public Health, Andalusian School of Public Health, Granada, Spain; ^2^Center for Biomedical Research in Epidemiology and Public Health Network, Carlos III Health Institute (ISCIII), Madrid, Spain; ^3^Department of Statistics and Operative Research, and Institute of Mathematics, University of Granada, Granada, Spain; ^4^Granada Biosanitary Research Institute, Granada, Spain; ^5^Department of Nursing, Faculty of Nursing, Physiotherapy and Podiatry, University of Seville, Seville, Spain

**Keywords:** COVID-19, surveys and questionnaires, mental health, non-probability surveys, reweighting

## Abstract

**Background:**

The use of health surveys has been key in the scientific community to promptly communicate results about the health impact of COVID-19. But what information was collected, where, when and how, and who was the study population?

**Objective:**

To describe the methodological characteristics used in large health surveys conducted in Spain early on in the COVID-19 pandemic.

**Methods:**

Scoping review. Inclusion criteria: observational studies published between January 2020 and December 2021, with sample sizes of over 2,000 persons resident in Spain. Databases consulted: PubMed, CINAHL, Literatura Latinoamericana y del Caribe en CC de la Salud, Scopus, PsycINFO, Embase, Sociological Abstracts, Dialnet and Web of Science Core Collection. We analyzed the characteristics of the literature references, methodologies and information gathered in the surveys selected. Fifty five studies were included.

**Results:**

Sixty percentage of the studies included had mental health as their main topic and 75% were conducted on the general adult population. Thirteen percentage had a longitudinal design, 93% used the internet to gather information and the same percentage used non-probability sampling. Thirty percentage made some type of sampling correction to reduce coverage or non-response biases, but not selection biases. Sixty seven percentage did not state the availability of their data.

**Conclusions:**

Consistent with the extensive use of non-probability sampling without any bias correction in the extraordinary setting created by COVID-19, quality population frameworks are required so that probability and representative samples can be extracted quickly to promptly address other health crises, as well as to reduce potential coverage, non-response and particularly selection biases by utilizing reweighting techniques. The low data accessibility despite the huge opportunity that COVID-19 provided for Open Science-based research is striking.

## Introduction

Health surveys are a fundamental support tool for decision-making in health planning. They provide information on magnitude, distribution and trends in health, the social factors that determine them and the use of social services from the population's perspective. They permit identification of the main challenges for prioritizing activity, designing and developing intervention strategies, evaluating and allocating resources, and the main risk groups in terms of health, lifestyles, and access to health services (1).

The highly significant role of surveys for Public Health was greater still with the COVID-19 pandemic due to the urgent requirement for its health impact outcomes to be conveyed (2). This context led the scientific community, regardless of location or area of expertise, to gather information about the pandemic quickly, and here surveys were the key tool. This resulted in the publication of an extremely large number of scientific articles mainly relating to population lockdown and restrictions on mobility (3–9); measures that brought changes and adaptations to the methods and techniques for collecting information through surveys.

In this respect, non-probability surveys conducted with volunteers via the internet proliferated: for example, via websites, mobile apps, and publicity on social media. These types of survey enable statistics to be accessed more rapidly and at the same time provide an inexpensive means of compiling data, although they are subject to selection and coverage biases. This does not happen with probability surveys, often used by health statistics services such as Gold Standard, since they enable valid inferences to be made about the population without having to include hypotheses in models (10, 11). Furthermore, sampling theory based on distribution of probability arising from sample design enables any potential sampling errors in the estimators concerned to be determined and controlled (11).

Prior statistical reweighting is therefore necessary in non-probability sampling in order to obtain valid and precise estimates that eliminate, or at last reduce, these biases (12, 13). In sum, the survey methodology used to compile and analyze information has a direct effect on the quality of the results obtained.

Finally, the use of health surveys has been key in the scientific community to promptly communicate results about the health impact of COVID-19. But what information was collected, where, when and how, and who was the study population? This research question justified the study objective of this work as the performance of a scoping review to describe the methodological characteristics of large health surveys conducted in Spain at the beginning of the COVID-19 pandemic.

## Methods

We performed a scoping review (14) using the methodological framework developed by Arksey and O'Malley (15) and the Joanna Briggs Institute (16), and reported in line with PRISMA-ScR guidelines (17). We based our scoping review following the Population, Concept and Context (PCC) format as the research review question (18). Thus, the research review question for the Population was “Spanish surveys,” for the Context was “COVID-19,” and for the Concept was “Survey Methodology.”

The following databases were consulted: PubMed, CINAHL (Ebscohost), Literatura Latinoamericana y del Caribe en CC de la Salud (LILACS), Scopus, PsycINFO (Proquest), Embase (Elsevier), Sociological Abstracts (Proquest), Dialnet and Web of Science Core Collection. We selected biomedical and multidisciplinary databases because most of the surveys during the pandemic were related to social services and according to the following criteria:

- Databases with large coverage and large numbers of journals included: Pubmed, Scopus, Embase.
- Databases with Spanish journals and articles in Spanish included: LILACS, Scopus, Dialnet.
- Databases specializing in health literature: Pubmed, CINAHL, Embase.
- Databases specializing in socio-sanitary literature: PsyINFO, Sociological Abstract, WOS, Scopus.

This search was complemented with gray literature information sources: OpenGray (unpublished literature), Gray Literature Report, the University of Oxford Global Directory for COVID surveys (https://supertracker.spi.ox.ac.uk/surveys/) and open searches in Google. The searches were developed between January 2020 and December 2021. These coverage dates of the databases were given by the novelty of the subject, COVID-19. There were no language restrictions. The search strategy was conducted through a combination of controlled terminology (MeSH/Emtree) and free language representative of the concepts COVID-19, surveys, and Spain, and was adapted to the different databases consulted (Table 1).

**Table 1:** Search terms (PubMed search strategy).

1. (“surveys and questionnaires” [MeSH Terms] OR “health surveys” [MeSH Terms] OR “healthcare survey” [Text Word] OR survey^*^ [Text Word] OR questionnaire^*^ [Text Word] OR interview^*^ [Text Word])
2. (“COVID-19” [All Fields] OR “COVID-19” [MeSH Terms] OR “COVID-19 vaccines” [All Fields] OR “COVID-19 vaccines” [MeSH Terms] OR “COVID-19 serotherapy” [All Fields] OR “COVID-19 serotherapy” [Supplementary Concept] OR “COVID-19 nucleic acid testing” [All Fields] OR “COVID-19 nucleic acid testing” [MeSH Terms] OR “COVID-19 serological testing” [All Fields] OR “COVID-19 serological testing” [MeSH Terms] OR “COVID-19 testing” [All Fields] OR “COVID-19 testing” [MeSH Terms] OR “sars cov 2” [All Fields] OR “SARS-CoV-2” [MeSH Terms] OR “severe acute respiratory syndrome coronavirus 2” [All Fields] OR “ncov” [All Fields] OR “2019 ncov” [All Fields] OR ((“coronavirus” [MeSH Terms] OR “coronavirus” [All Fields] OR “cov” [All Fields]) AND 2019/11/01:3000/12/31[Date - Publication]))
3. “Spain” [Text Word]
4.1 AND 2 AND 3

The results were transferred to a Mendeley database, subsequent to which we identified and classified articles on the Rayyan web platform, eliminating duplicate references (19). Initial selection was performed by peers (ACL, EM, AO, CSC, and DY) through screening titles and abstracts for eligibility. In the event of disagreement, a third researcher was asked to arbitrate.

Inclusion criteria were observational studies published between January 2020 and December 2021, with a total effective sample of ≥2,000 persons resident in Spain, published in English and Spanish. Exclusion criteria were studies that did not collect any information on perception of physical or mental health, qualitative, intervention or experimental studies and studies based on records. In the event of several articles stemming from the same survey, the one providing the most information about the survey was selected.

Data were extracted independently (by CSC and DY) using a standardized, predefined form that included variables relating to characteristics:

- Literature references: link to publication, first author institution of work, date of publication, language, name of journal, type of publication (scientific article, report, review, comment, letter), open access (yes/no), impact factor and position (highest quartile) (20).
- Survey: geographical area, study population, study design, sampling design, effective sampling size, sample weighting and other corrections, survey type, date information collected, response rate, waves or measurements, analyses performed, availability of microdata (Tables 1, 2).
- Information collected: objective of study, primary topic [defined as mental health (43), lifestyle habits (27), wellbeing (76), quality of life (29), life satisfaction (42), perceived risk of infection (56), resilience (45) and working conditions (22)], information blocks, scales/composite variables, conclusions, observations (Supplementary Tables 1, 2).

**Table 2:** Methodological characteristics of the selected surveys.

**Reference**	**Geographic scope (number of countries)**	**Study population**	**Study design**	**Effective sampling size**	**Sampling adjustments**	**Field work (start date)**	**Analysis performed**
Ahrendt et al. (21)	Countries (22)	General population (≥18)	Cross-sectional^**^	2,000–5,000	Correction factor	April 2020	Descriptive
Ajanovic et al. (23)	Spain	General population ( ≤ 16)	Cross-sectional	2,000–5,000	N/A	July 2020	Bivariate
Alonso et al. (24)	Region (6)	Healthcare professionals	Longitudinal	5,000–10,000	Calibration	May 2020	Logistic models
Arpino et al. (25)	Countries (3)	General population (≥18)	Cross-sectional	2,000–5,000	Post-stratification	April 2020	Descriptive
Carpintero-Rubio et al. (26)	Spain	General population (≥18)	Cross-sectional	2,000–5,000	N/A	May 2020	Bivariate
Cervera-Martínez et al. (27)	Spain	General population (≥18)	Cross-sectional^*^	5,000–10,000	N/A	April 2020	Linear models
Codagnone et al. (28)	Countries (3)	General population (≥18)	Cross-sectional^***^	2,000–5,000	Post-stratification	April 2020	Random forest models
Coronado et al. (29)	Spain	Women 40–70	Cross-sectional	2,000–5,000	N/A	April 2020	Linear models
de Pedraza and Vicente (30)	Spain	General population	Cross-sectional	2,000–5,000	Correction factor	March 2020	Logistic models
Centre d'Estudis d'Opinió (CEO) (31)	Region	General population (≥16)	Cross-sectional	10,000–50,000	Correction factor	April 2020	Descriptive
Faris et al. (32)	Spain	General population	Cross-sectional	2,000–5,000	Post-stratification	May 2020	Tobit models
Farres et al. (33)	Region	General population (≥16)	Cross-sectional	>50,000	N/A	April 2020	Bivariate
Fernández-Prados et al. (34)	Spain	General population (≥18)	Cross-sectional	2,000–5,000	N/A	June 2020	Logistic models
Garcia-Adasme et al. (35)	Region	General population ( ≤ 16)	Cross-sectional	2,000–5,000	N/A	April 2020	Bivariate
García-Álvarez et al. (36)	Spain	General population (≥18)	Cross-sectional	10,000–50,000	N/A	March 2020	Logistic models
García-Dantas et al. (37)	Spain	General population (≥18)	Cross-sectional	2,000–5,000	N/A	March 2020	Bivariate
Garcia-Esquinas et al. (38)	Spain	General population (≥65)	Longitudinal^**^	2,000–5,000	N/A	April 2020	Mixed models
Garrido-Cumbrera et al. (39)	Spain	General population (≥16)	Cross-sectional	2,000–5,000	N/A	April 2020	Logistic models
Gómez-Salgado et al. (40)	Spain	General population (≥18)	Cross-sectional	2,000–5,000	N/A	March 2020	Logistic models
Gonzalez et al. (41)	Spain	General population (≥18)	Cross-sectional	2,000–5,000	N/A	March 2020	Bivariate
Gonzalez-Bernal et al. (42)	Spain	General population (≥18)	Cross-sectional	2,000–5,000	N/A	March 2020	Linear models
Gonzalez-Sanguino et al. (43)	Spain	General population (≥18)	Longitudinal^**^	5,000–10,000	N/A	March 2020	Linear models
Grané et al. (44)	Countries (45)	General population (≥50)	Longitudinal^*^,^***^	10,000–50,000	Calibration	June 2020	Cluster
Hidalgo et al. (46)	Spain	General population (≥18)	Cross-sectional^***^	5,000–10,000	N/A	April 2020	Bivariate
Jacques-Aviñó et al. (47)	Spain	General population	Cross-sectional	5,000–10,000	N/A	April 2020	Logistic models
Jané-Llopis et al. (48)	Region	General population (≥16)	Cross-sectional	10,000–50,000	N/A	April 2020	Linear models
Jones (49)	Countries (50)	General population	Longitudinal^**^	10,000–50,000	Correction factor	March 2020	Descriptive
Justo-Alonso et al. (51)	Spain	General population (≥18)	Cross-sectional	2,000–5,000	N/A	March 2020	Bivariate
Kim and Ryu (52)	Countries (25)	General population	Cross-sectional	2,000–5,000	N/A	March 2020	Mixed models
Lázaro-Pérez et al. (53)	Spain	Armed forces professionals	Cross-sectional	2,000–5,000	N/A	August 2020	Logistic models
López-Bueno et al. (54)	Spain	General population (≥18)	Cross-sectional	2,000–5,000	N/A	March 2020	Logistic models
Maestro-Gonzalez et al. (55)	Spain	General population	Cross-sectional	5,000–10,000	N/A	March 2020	Multivariate analysis (N/A)
Mansilla Domínguez et al. (56)	Spain	General population (≥18)	Cross-sectional	10,000–50,000	Post-stratification	March 2020	Logistic models
Martin et al. (57)	Spain	Healthcare professionals	Cross-sectional	2,000–5,000	N/A	April 20	Linear models
Martinez-Bravo and Sanz (58)	Spain	General population (≥18)	Cross-sectional^*^	2,000–5,000	Correction factor	May 2020	Descriptive
Méndez-Giménez et al. (59)	Spain	General population (≥16; < 92)	Cross-sectional	2,000–5,000	N/A	March 2020	Logistic models
Miranda-Mendizabal et al. (60)	Spain	General population	Cross-sectional^*^	2,000–5,000	Correction factor	October 2020	Logistic models
Morales-Vives et al. (61)	Spain	General population (≥18)	Cross-sectional	2,000–5,000	N/A	March 2020	Bivariate
Oliver et al. (62)	Spain	General population	Cross-sectional	>50,000	Correction factor	March 2020	Logistic models
Viejo et al. (45)	Spain	General population	Cross-sectional	2,000–5,000	N/A	October 2020	Mixed models
Pérez-Raya et al. (22)	Spain	Healthcare professionals	Cross-sectional	10,000–50,000	Correction factor	April 20	Descriptive
Pinedo et al. (6)	Spain	General population	Cross-sectional	2,000–5,000	N/A	March 2020	Structural Equation models
Planchuelo-Gómez et al. (63)	Spain	General population	Longitudinal^*^	2,000–5,000	N/A	April 2020	Mixed models
Pouso et al. (64)	Countries (9)	General population	Longitudinal	5,000–10,000	N/A	April 2020	Mixed models
Rodríguez-Barranco et al. (65)	Spain	General population	Cross-sectional	2,000–5,000	N/A	April 2020	Logistic models
Rodríguez-Larrad et al. (66)	Spain	University students	Cross-sectional	10,000–50,000	N/A	April 2020	Bivariate
Rodríguez-Pérez et al. (67)	Spain	General population	Cross-sectional	5,000–10,000	N/A	March 2020	Multivariate analysis (N/A)
Rodriguez-Ruiz et al. (68)	Spain	Healthcare professionals	Cross-sectional^*^	2,000–5,000	N/A	October 2020	Bivariate
Romero et al. (69)	Spain	Healthcare professionals	Cross-sectional	2,000–5,000	N/A	April 2020	Bivariate
Salas-Nicás et al. (70)	Spain	General population	Cross-sectional	10,000–50,000	Correction factor	April 2020	Bivariate
Sánchez-Cantalejo et al. (71)	Region	General population (≥16)	Longitudinal^**^,^***^	10,000–50,000	Calibration, Propensity Score Matching, Machine Learning	April 2020	Mixed models
Valiente et al. (72)	Spain	General population	Cross-sectional	2,000–5,000	N/A	April 2020	Logistic models
Vall-Roqué et al. (73)	Spain	Women (14–35)	Cross-sectional	2,000–5,000	N/A	May 2020	Hierarchical models
Villanueva et al. (74)	Spain	General population (≥18; < 65)	Cross-sectional	2,000–5,000	Correction factor	April 2020	Bivariate
Yélamos Agua et al. (75)	Spain	Chronic patients	Cross-sectional	2,000–5,000	N/A	April 2020	Logistic models

^*^Survey with 2 measurements; ^**^survey with 3 or more measurements (Jones, SP collects 29 measurements with COVID data); ^***^probabilistic sample; N/A, not available in the manuscript.

The variables of the second paragraph (survey characteristics) were selected from the STROBE (50) list, given that the studies in this review are observational.

## Results

**Figure 1:**
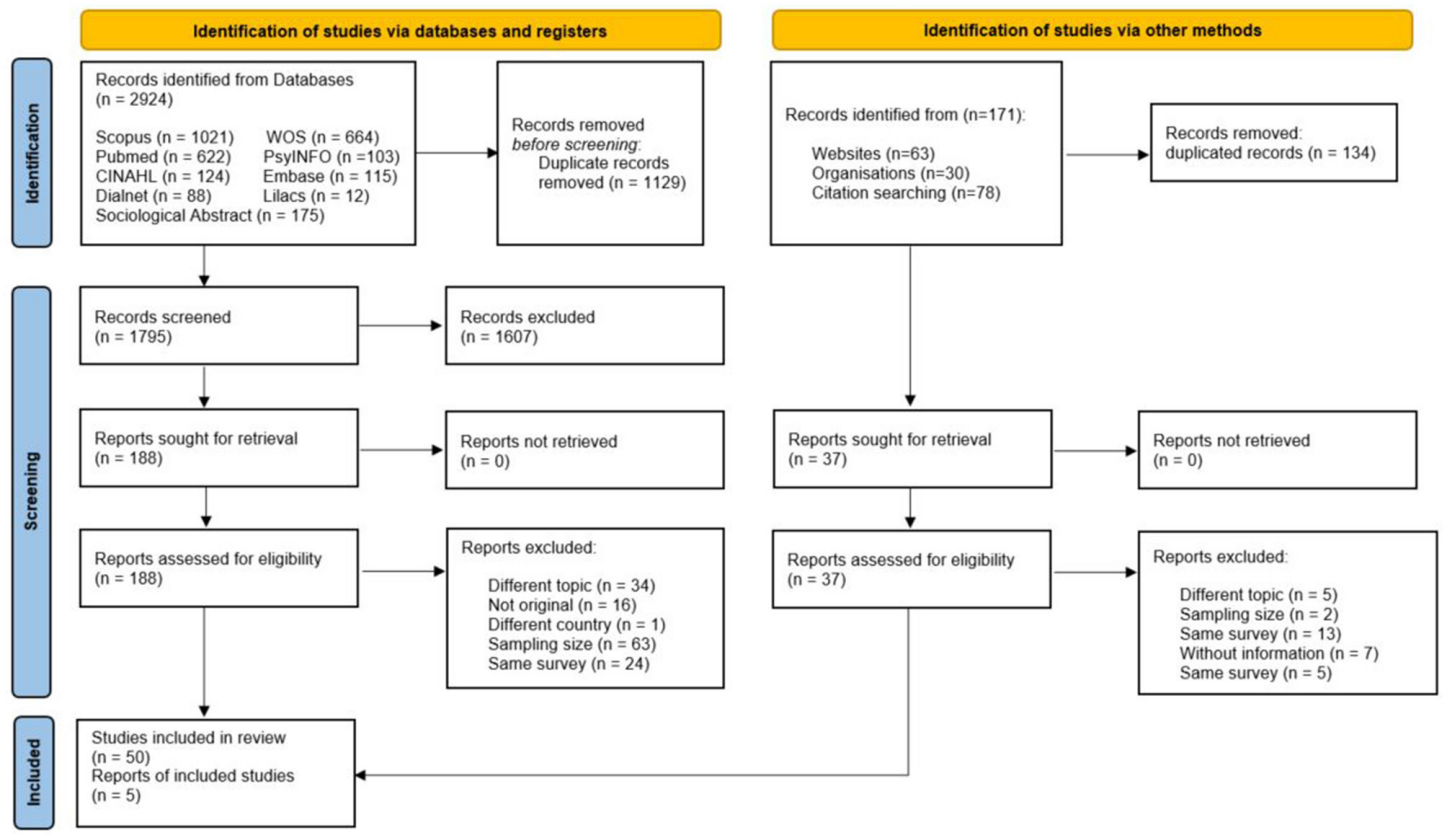
PRISMA 2020 flow diagram for new systematic reviews which included searches of databases, registers, and other sources (77). For more information, visit: http://www.prisma-statement.org/.

A total of 3,095 articles were identified following the search strategy described above. Two thousand nine hundred twenty-four articles were identified using scientific literature databases and 171 using gray literature. A full-text check was performed on 225 of them, i.e., 6.4 and 21.6%, respectively, for scientific literature databases and gray literature. Finally, 55 references were included for the analysis (Figure 1).

Table 2 shows the methodological characteristics of each survey selected. The majority were signed by first authors from Spanish institutions (88%), 76.4% focused on Spain, 10.9% were conducted in smaller geographical units such as Autonomous Communities or municipalities, and 12.7% in various countries (in addition to Spain).

Almost half of the surveys selected published their results in 2020 (45%) and all of them began field work in 2020, one third of them in March (32.7%), 78.2% during the lockdown (March to April 2020) and 90.9% during the first state of emergency (March to June 2020). In addition, 80% of surveys collected information on one occasion or through one measurement. The YouGov bi-weekly information study (49) was found to have collected data on COVID-19 on 29 occasions.

As regards the study population of the 55 surveys selected for the analysis, 74.5% of them addressed the general adult population as their study population, while 9.1% considered the healthcare professionals (22, 24, 57, 68, 69). The same percentage of studies (3.6%, two surveys) considered as the study population the pediatric population (23, 35), women (29, 73) or people aged above 50 years old (38, 44). We also found one survey on chronic patients (75), on people aged over 50 or 65 years old, on the university community and on armed forces professionals.

The main topics among the selected surveys were mental health (60.0%), lifestyle habits (10.9%), wellbeing (7.3%), and quality of life, life satisfaction, perceived risk of infection, resilience and working conditions (3.6%). Information regarding the objectives, information blocks and scales or composite variables was also gathered and is available in Supplementary Tables 1, 2.

**Figure 2:**
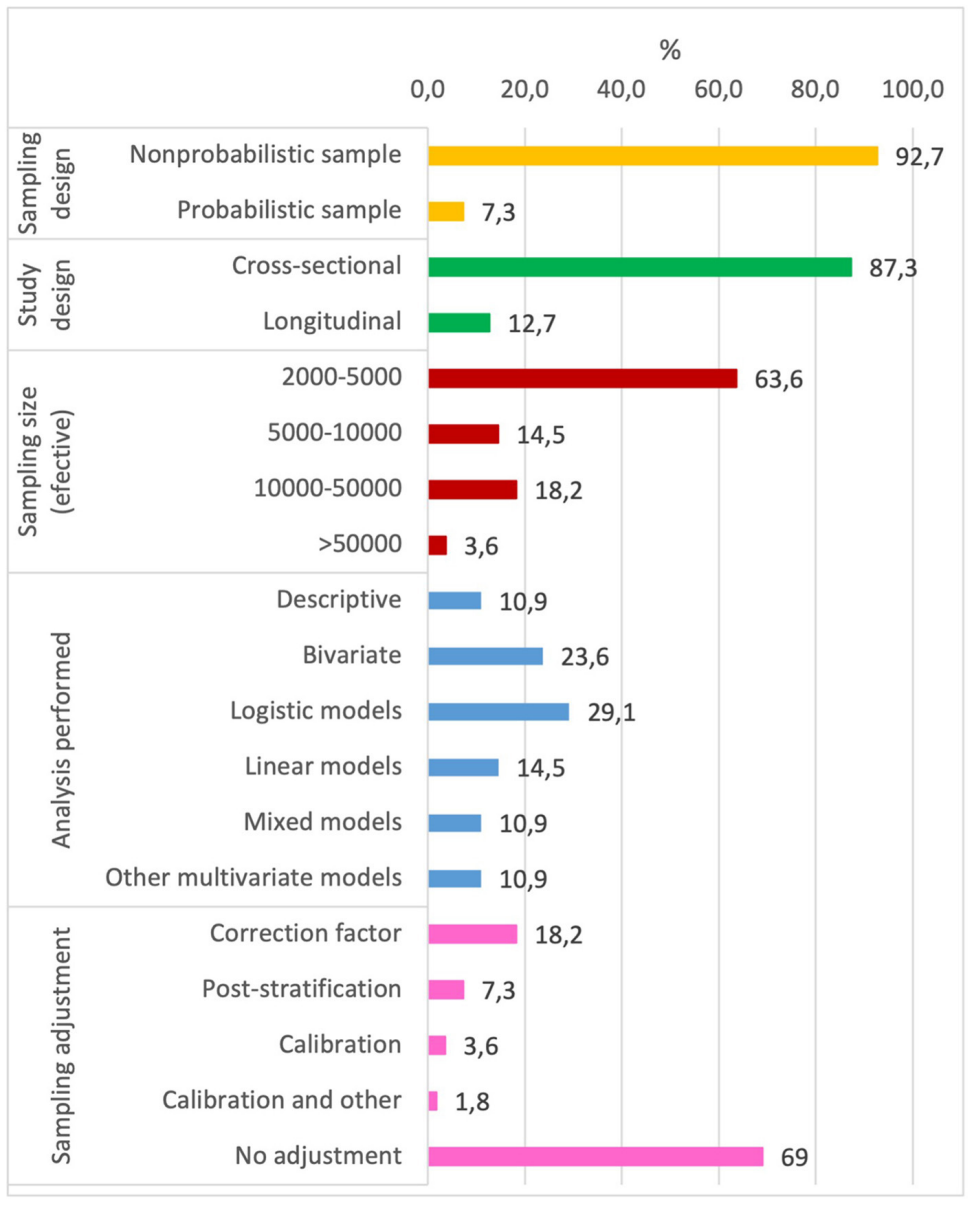
Sample design and statistical analysis of the selected surveys.

As regards sampling design, four of the fifty-five surveys selected (7.3%) had a probability design (28, 44, 46, 71) and seven (12.7%) were longitudinal surveys (Figure 2) (24, 38, 43, 44, 49, 63, 78), one on healthcare professionals (24) and the rest on the general population. Furthermore, three of these seven longitudinal surveys were cohort studies predating the pandemic (38, 44, 49). 92.7% of the surveys selected for the analysis gathered their data through online surveys, e.g., Qualtrics, Google forms, Lucid, SurveyGizmo or Surveymonkey, and 7.3% by telephone.

In respect of sampling size, 35 surveys had between 2,000 and 5,000 participants (effective sample), two being found with over 50,000 participants (33, 62), both of which were online cross-sectional surveys. Additionally, 92.7% of the surveys included did not report the response rate.

As regards the statistical analysis conducted, thirty-six surveys developed a multivariate model, the most frequent being binary logistic (16), linear (eight surveys) and mixed (six surveys). Other multivariate models used were multi-level (73), cluster (44), principal components (32), random forest (28) and structural equations (6).

The distribution of groups of observations in the health surveys usually differs from the distribution in the survey population due to several reasons (coverage of the sampling frame, sample design, or patterns of unit non-response). Weighting is one of the best ways to reduce variances and to correct for frame deficiencies. In that sense, 30% implemented some type of sampling adjustment (Figure 2). The most frequent correction was of sample representativeness in view of sociodemographic variables using records or reference surveys (ten surveys). Post-stratification and calibration were applied only in four and two surveys, respectively. These methods are usually considered in official governmental surveys to minimize errors associated with incomplete sampling frames and with sampling non-response (79–81). Of note is the Health and Social Survey (71) which, in addition to calibration to reduce potential coverage or representativeness biases, implemented other methods based on Propensity Score Matching and Machine Learning to reduce biases due to lack of response in longitudinal samples. No voluntary or non-probability surveys were identified that used correction to reduce the selection bias concerned.

Lastly, most of the surveys included (67.2%) did not report on the availability of microdata.

## Discussion

As far as we are aware, this is the first scoping review on health surveys relating to COVID-19 and their main methodological characteristics; actually, we found only one similar study dating from 2013 (82), albeit based on population health surveys conducted at Autonomous Community level in Spain and, most relevantly, without the extraordinary context provided by the pandemic, in which there was an urgent need to gather data to support timely evidence-based decisions. Moreover, searching in so many bibliographic resources is a strength of this scoping review.

Our main purpose with this review was to describe the methodological characteristics of surveys conducted early on in the pandemic, hence the search was focused on 2020 and 2021, noting that all surveys started during the first year of COVID-19. In fact, four out of every five surveys (78.2%) were conducted during the 2 months of the first lockdown period (March and April 2020). This demonstrates the rapid response by and considerable effort that the scientific community invested in attempting to provide information about the impact of COVID-19 on the population's health, with particular emphasis on mental health evidenced by the fact that more than half of the surveys (60%) focused on this as their main topic. This response was possible thanks to the internet: nine out of every ten surveys (92.7%) used social media, media sampling to recruit participants, or online subscription panels via this channel. The use of these types of survey expanded to such an extent during COVID-19 lockdowns that, along with more social considerations such as increasingly widespread internet access and use, they took over from traditional survey methods. In this regard, our study found that only four of the fifty-five surveys reviewed were conducted over the phone (7.3%) and, as was to be expected, no face-to-face surveys were identified.

However, despite the efforts made by official statistical institutions, for example the European Statistical System through its Quality Assurance Framework (83), the scientific community faced the difficulty of obtaining quality population frameworks from which quickly to extract probability samples representative of the study populations concerned. As our review shows, 92.7% of the surveys were based on non-probability sampling, which confirms their extensive use in the extraordinary setting created by COVID-19. Given the rapid inclusion of these types of study, we could ask ourselves the following question in relation to official health statistics: are probability surveys destined to disappear? In Beaumont's opinion (84), this moment has not yet arrived because the alternatives are not reliable and general enough to eradicate the use of probability surveys without having a deleterious effect on the quality of estimates.

Non-probability surveys present two advantages: they can collect large samples and they can do this in a short period of time. This is evidenced in our review, which shows that one out of every five surveys (21.8%) had a sample size of over 10,000 people, bearing in mind that one of the inclusion criteria was having an effective sample size of over 2,000. By contrast, the main drawback of non-probability surveys is that they present significant issues in terms of selection and coverage biases, thus compromising the generalization of results to the study population (85). Our review found that 30.9% of the surveys conducted implemented some type of sampling adjustment by means of correction factors, post-stratification sampling weighting, or calibration with sociodemographic variables such as sex, age or geographical area based on records or reference surveys. However, these adjustments do not correct volunteer bias (86), shown by the fact that we did not find any surveys that included non-probability selection of the people surveyed in their estimates. In this respect, different reweighting techniques have been developed in recent years using Propensity Score Adjustment, Statistical Matching, Kernel Weighting and combinations of these techniques (13, 79, 87–89) that have shown themselves to be highly effective for eliminating biases and increasing representativeness in non-probability surveys.

Despite these limitations, non-probability sampling can complement probability sampling if it is designed as a means to offset known biases in probability sampling by focusing on survey participant profiles that tend to be under-represented in such surveys (90). This notwithstanding, we did not find it being used in our review. Furthermore, non-probability surveys can be useful in some cases for providing relevant information that would not otherwise be available, for example in studies on small sub-populations where probabilistic sampling will encounter problems in fulfilling sample size requirements, good access to the study population or a suitable population framework for sample selection (91). However, here again we did not find it being used in our review, because the majority of surveys in Spain on the health impact of COVID-19 were conducted on the general adult population (74.5%). Nor did we identify any studies on more potentially vulnerable populations such as ethnic minorities, residents in care homes for the older adult or in deprived areas, other than the Health and Social Survey which, in addition to conducting surveys on the general population, also collects data on populations living in deprived areas (71). This percentage of general population surveys could be even larger, given that we eliminated forty-two studies stemming from the same survey. It must be noted that this probability survey was able to be conducted through the construction of a population framework during COVID-19 based on linking population records (92) and social records (93). In addition, the interviews in it were conducted not via the internet but rather by telephone, a more suitable channel for reaching these types of population given the continuing digital gap. So population frameworks such as this one provide opportunities for conducting other probability surveys (by telephone or in person) on these types of population.

Another outcome of our review worth noting is the low proportion of longitudinal surveys identified (12.7%). Surveys repeated over time are more difficult to conduct and analyze, but they do permit evaluation of changes in study variables in the same population, a key area for being able to obtain an overview of the pandemic and not just of its characteristics at a given moment in time (94). A sampling design that has proved useful in social research is rotating panel surveys where there is partial renewal of units (to mitigate panelist fatigue), the main advantage of which is that both cross-sectional and longitudinal estimates can be made (71), overcoming the potential limitation of many longitudinal studies in terms of needing to have rapidly available information on the state of the population. However, none of the surveys identified in our review used this design, other than the Health and Social Survey set up at the beginning of the COVID-19 state of emergency (71). This means that many of the surveys identified do not permit the changing effect of the pandemic on health in a single population to be known. Moreover, they were conducted at a very specific moment in time in highly exceptional circumstances, which must also be taken into account when extrapolating their results.

Lastly, this review is in line with other studies that show the high volume of scientific output related to COVID-19 (95). In our case, we identified more than 3,000 studies performed in Spain over 2 years, of which we selected 1.8% (55 surveys) for our review. Additionally, although our review centers on Spain, the studies it includes have a large international component given that 12.7% of them looked at other countries (some more than 27) (21, 25, 52, 64, 96) and 58.2% of them were published in journals situated in the first quartile (Journal Citation Reports).

As regards the search and the record created, they enable other analyses to be performed in subsequent years on specific topics such as mental health, and studies without a given exclusion criterion to be easily retrieved (thus enabling the analysis performed in this review to be repeated in other studies). For example, we considered as the last exclusion criteria surveys with a sample size of < 2,000 individuals. Our objective was to select large health surveys in terms of guaranteeing that sampling errors in overall estimates were below three percentage points assuming *p* = q = 0.5, 95% confidence level (power level did not apply because we considered observational studies), 0% sample loss because we refer to effective sample (not the theoretical one), and design effect two. If a lower sample size were required, it would be very easy to retrieve those studies through Rayyan and repeat the analysis. However, although our record facilitates identifying these studies through Rayyan, it is worth pointing out that barely one third of the surveys reviewed make their data openly available, and this hinders performing these studies or other analyses such as, for example, reweighting techniques which would provide more reliable estimates. This clearly reflects the ongoing lack of research based on Open Science (97), despite the major opportunity provided by COVID-19 to reverse this situation (98).

## Author contributions

Conceptualization and funding acquisition: AC-L, MR, and CS-C. Methodology: AC-L, CS-C, AO-d-L-L, EM-R, and CH-C. Data curation and analysis: AC-L, CS-C, DY, AO-d-L-L, and EM-R. Writing—original draft preparation: AC-L, CS-C, and DY. Writing—review and editing: MR, AC-L, CS-C, AO-d-L-L, EM-R, and CH-C. Project administration: AC-L. All authors have read and agreed to the published version of the manuscript.
