# Supplementary material for: Scoping review of the methodology of large health surveys conducted in Spain early on in the COVID-19 pandemic
Source: Front Public Health. 2023 Aug 2;11:1217519. doi: 10.3389/fpubh.2023.1217519 (PMC10438850; doi:10.3389/fpubh.2023.1217519)
Supplement: Supplementary file 1 [file Data_Sheet_1.pdf]

Supplementary Tables.

Supplementary Table 1. Objectives, information blocks and scales of included studies.

**First author**	**Objective/s**	**Information blocks**	**Scales/composites variables (Abbreviations in Supplementary Table 2)**
Ahrendt, D	To investigate the impact on well-being, health, safety, work, telework, work–life balance and financial situation	Well-being, health, work-life balance, employment and remote work, trust in institutions, supporting measures, social trust, vaccination	EQLS, EWCS, EU-SILC
Ajanovic, S	To understand the perceived impact of lockdown measures on the mental health and well-being of minors	Sociodemographic variables, household, risk perception and transmission knowledge, coping with confinement, biorhythms, emotional distress, use of electronic devices and social media, satisfaction with remote schooling	Coping with confinement scale, satisfaction with remote schooling scale
Alonso, J	To estimate the prevalence of specific mental disorders in healthcare professionals and in subsamples with/without prior mental disorders; and to estimate associations of individual and professional characteristics, COVID-19 infection status, and COVID-19 exposure	Sociodemographic variables, COVID-19 exposure, infection status	PHQ-8, GAD-7, an item from WMHICS for panic attacks, PCL-5, CAGEAID, Sheehan Disability Scale, CIDI
Arpino, B	To better understand the indirect consequences of the COVID-19 outbreak	Intergenerational relationships, living arrangements, mental health, events experienced during the lockdown, intentions for the future 3 years	Risk and time preferences scale
Carpintero-Rubio, C	To describe the perception of musculoskeletal pain in the population and how the state of confinement has interfered with it, as well as identifying the sociodemographic, occupational, physical, and psychosocial factors involved	Sociodemographic variables, pain and related factors, use of electronic devices; sedentary behaviour and physical activity, perception of restlessness, impatience, fatigue, concentration, irritability/fatigue, sleep disorders, and concern about symptoms	VAS
Gonzalez Casas, D	To evaluate the effect of confinement on depressive mood in Spanish residents during the state of emergency. And to analyse the impact of sex, age, size of dwelling and income	Sociodemographic variables and house size	CES-D scale
Cervera-Martínez, J, Balanzá-Martínez V	To compare self-reported changes on lifestyle behaviours during two phases of the pandemic in Spain, and to evaluate clinical and sociodemographic factors associated with lifestyles	Sociodemographic variables, nutrition, physical activity, sleep, people living in the house, self-isolation, COVID-19 diagnosis, loss of significant ones, SRH, change in lifestyle behaviours during the pandemic, health problems, PHQ-2	SMILE-C scale, PHQ-2, AUDIT-C
Codagnone, C	To examine the elicitation of citizens' concerns over the downplaying of the economic consequences of the lockdown. To predict the level of stress, anxiety and depression associated with being economically vulnerable and being affected by a negative economic shock	Sociodemographic variables, economic variables, stress, anxiety, depression, COVID19 exposure	Depression, Anxiety and Stress Scale and Stanford Acute Stress Reaction Questionnaire
Coronado, P J	To assess the impact of confinement due to the pandemic on HRQoL and resilience in peri- and postmenopausal women	Sociodemographic variables, Cervantes-SF and RS-14 scales	HRQoL and RS-14 scales
de Pedraza, P	To study the effect of COVID-19 NPIs on individuals' subjective well-being using Spanish regions as a case study and	Sociodemographic variables, bars closure, self-assessed health status, living with a partner, children, employment situation, exercise, job loss,	SATLIFE

First author	Objective/s	Information blocks	Scales/composites variables (Abbreviations in Supplementary Table 2)
	exploring the relationship between bars and restaurant closures and life satisfaction	loneliness, expectations of income loss, family or friends diagnosed with COVID-19	
Centre d'Estudis d'Opinió (CEO)	To report the impact of the COVID-19 health crisis on the perceptions and opinions of Catalan society	Socioeconomic variables, perception and conditions of confinement, economic impact, health impact, political opinion	Scale on the impact of the pandemic on health and economy
Faris, LH	To test whether substance use disorder is a risk factor for depressive symptoms, secondly, to determine if social support is a protective factor for depressive symptoms, and finally, to examine the moderating effect of social support on the association between substance use disorder and depressive symptomatology.	Sociodemographic variables, depressive symptoms, health-related variables, social support, substance use disorder during the last month	PHQ-8, OSSS-3
Farres, J	To evaluate the impact of COVID-19 on the psychosocial sphere in both the general population and healthcare workers (HCWs).	Sociodemographic variables, habits, and COVID-19-related health status during confinement	Socioeconomic precariousness index, depression index, anxiety index, stress index or PTSD
Fernández-Prados, JS	To discover the factors which would allow the Spanish population to positively cope with or tolerate a prolongation of the state of emergency and lockdown	Sociodemographic variables, ideology, and religiosity	None
Garcia-Adasme, SI	To determine the impact of COVID-19 related home confinement on the paediatric population by focusing on anxiety, behavioural disturbances, and somatic symptoms	Child age and gender, and information on the family environment related to COVID-19	Children's Manifest Anxiety Scale's
García-Álvarez, L	To describe the early impact of the pandemic and lockdown on mental health and to explore the factors that may contribute to or mitigate the impact in a large sample of the Spanish population. Furthermore, to compare the psychological impact according to absence or presence of past / current mental disorders	Sociodemographic variables, income, changes in work and income due to COVID-19, number and age of dependent children, and dependent older adults, activities during lockdown, health conditions, past / current mental problems, COVID-19 variables	DASS-21 (Spanish version), and the IES
García-Dantas, A	To evaluate the psychological responses to the first wave of the pandemic in Spain	Sociodemographic variables, household composition and housing location, physical and mental state perception, psychiatric illness, routines and habits during quarantine, exposure, perceived threat of COVID-19, somatization, agoraphobia, sleep patterns, drug use, hypochondriacal concerns, and isolation	IES-R, DASS-21, SCS
Garcia-Esquinas, E	To describe the main changes in health behaviours and in mental and physical health between a pre-pandemic period and weeks 7 to 15 after the beginning of the COVID-19 lockdown among participants in four cohorts of community-dwelling older adults in Spain	Sociodemographic variables, health behaviours, mental and physical health, housing conditions, aging experiences, social isolation, tobacco consumption, MEDAS, physical activity, EPAQ, EEPAQ, the Global Physical Activity Questionnaire, PASE, weight and height, BMI, night-time sleep, quality of life SF, WHODAS, EuroQol-5D in TSHA and EXERNET, and chronic conditions	MEDAS, EPAQ, EEPAQ, PASE, BMI, quality of life SF, WHODAS, EuroQol-5D, EXERNET

First author	Objective/s	Information blocks	Scales/composites variables (Abbreviations in Supplementary Table 2)
Garrido, M	To provide an international perspective to understand the impact of confinement, due to the COVID-19 pandemic, on well-being and mental health	Sociodemographic varibles, housing, habits before and after confinement, health and well-being, diseases	WHO-5
Lázaro-Pérez, C	To know the level of anxiety in the face of death in these professionals; and, to determine the predictive variables in this phenomenon	Sociodemographic variables, perceptions of the current situation at work, and MBI subscales	Collett–Lester Death Anxiety Scale
Gómez-Salgado, J	To analyse the psychological distress in a Spanish population sample during the COVID-19 pandemic	Sociodemographic variables, COVID-19 symptoms, health status, contacts, or exposure to COVID-19	GHQ-12
Gonzalez-Bernal, J	To study the association between perceived discrimination and mental health outcomes among health workers during the initial COVID-19 outbreak	Sociodemographic and COVID-19-related information	SWLS
Gonzalez-Sanguino, C	To conduct a longitudinal analysis of the effects of the pandemic and alarm situation on the mental health of the general population at three points in time	Sociodemographic and psychological variables	PHQ-2, GAD-2, PCL-C-2, InDI-D, UCLA-3, EMAS, FACIT-Sp12 and SCS
Grané, A	To analyse the effect of a pandemic shock on the well-being of the European population aged 50 or over	Demographic variables, physical and mental health, health care, employment and pensions, housing, household income, consumption, activities and expectations	Self-perceived health scale, EURO depression scale, life satisfaction scale
Hidalgo, MD	To analyse the psychological impact of the pandemic and the lockdown in the Spanish population and to identify what population profiles were most affected	Sociodemographic variables and psychological changes	Scale for psychological changes
Jacques-Aviñó, C	To assess the relations between social impact and mental health among adults living in Spain during COVID-19 lockdown measures, taking a gender-based approach into account	Social impact (socioeconomic status, living conditions, COVID-19 experiences and health-oriented behaviour) and mental health	GAD-7, PHQ-9
Jané-Llopis, E; Anderson, P	To report the impact of confinement on mental health and addictions, the link between reported length of confinement and severity, and detail the risk and protective factors that can provide insight for intervention preparedness in view of forthcoming partial or total confinements	Living condition, coping strategies, risk factors, individual, socio-economic concerns	PHQ-8, GAD-7, WEMWBS (short version), Catalan Health Survey, AUDIT-C, European Model Questionnaire for tobacco use, frequency of use of hypnosedatives, and use of drugs, OSSS-3
Jones, SP	To enable researchers to carry out their own analyses and develop novel findings.	Socioeconomic variables, preventative behaviours, trust in government's handling and COVID-19 vaccines, testing, symptoms, self-isolating, ability, and willingness to isolate, household variables, health conditions, and working status	Working status and the date of the survey response
Justo-Alonso, A	To explore, in specific age ranges, the mental health state of a Spanish community sample in the early stages of the pandemic	Sociodemographic and health variables, physical and mental states perceptions, psychiatric illness, routines, habits during the quarantine, concerns regarding the pandemic, exposure, perceived threat of COVID-19 infection, isolation, somatization,	DASS-21 and IES-R

First author	Objective/s	Information blocks	Scales/composites variables (Abbreviations in Supplementary Table 2)
		claustrophobia, sleep patterns and hypochondriac concern	
Kim, HH; Ryu, J	To analyze global health consequences of COVID-19 by focusing on the roles of normative beliefs on social distancing and country-level factors	Sociodemographic variables, adherence with COVID-19 containment measures, first-order injunctive beliefs, second-order injunctive beliefs, perceptions of government and public response, and mental health scales	PHQ-9
López-Bueno, R	To investigate changes in HRBs with confinement	Screen exposure, physical activity, fruit ang vegetable consumption, sleep time, alcohol consumption, smoking habit	PAVS short version
Maestro-Gonzalez, A	To analyse the quality of sleep of the Spanish population during the lockdown due to COVID-19	Sociodemographic variables, PSQI, occupational, health and sleep quality variable	PSQI
Mansilla Domínguez, JM	To analyse the perception of risk of COVID−19 infection in the Spanish population	Sociodemographic variables, protective measures, characteristics of the experience, health status during confinement, and risk perception	Affect index
Martin, J	To analyse the mental health and HRQoL of a sample of HCWs in Spain and to identify potential factors that have a significant effect on their mental health and HRQoL	Sociodemographic variables, COVID-19-related clinical data, and psychological support	GAD-7, PHQ-9, IES-R, ISI, and EQ-5D
Martinez-Bravo	To study consequences of the pandemic on Spanish households and levels of psychological well-being	Sociodemographic variables, economic variables, and subjective well-being	Self-reported well-being
Méndez-Giménez, A	To examine dose-response relationships between PA components and depressive symptoms during confinement, and to identify the optimal levels of PA to mitigate notable depressive symptoms (NDS)	PA components and depressive symptoms	IPAQ short form (SF), six-item self-report scale developed by Kandel and Davies (Spanish version)
Miranda-Mendizabal, A	To assess the socio-economic and psychological impact of the pandemic and lockdown in a representative sample of non-institutionalized Spanish adult population, and estimate the incidence of mental health disorders, including suicidal behaviours, and possible related factors.	Sociodemographic variables, health status, mental health, employment conditions and status, material deprivation, use of healthcare services, intimate partner violence and resilience	Not applicable
Morales-Vives, F	To determine which personal sociodemographic and psychological variables are related to adapting to lockdown in a Spanish population	Sociodemographic variables and psychometric measures	Big Five Inventory; Connor-Davidson Resilience Scale; Subjective Happiness Scale; Satisfaction With Life Scale, GHQ12, COVID-19 questionnaire
Oliver, N	To assess the situation and perception of the Spanish population in four key areas related to the COVID-19 pandemic: social contact behaviour during confinement, personal economic impact, labour situation, and health status	Sociodemographic variables, housing, social contact in the last 2 weeks, economic impact of the pandemic, workplace, labour situation, and health status	None
Perez viejo, JM	To analyse to what extent health, social and psychological factors are related to	Sociodemographic variables, confinement, employment, symptoms of COVID, deaths due to	Resilience scale

First author	Objective/s	Information blocks	Scales/composites variables (Abbreviations in Supplementary Table 2)
	resilience during the pandemic in the Spanish population	COVID, disability, illnesses, resilience, sadness, fear, aggressiveness, despair, tiredness, anxiety, feeling of no control, irritation, anger and worry about the future	
Pérez-Raya, F	To carry out an opinion survey of nurses about their working conditions at the beginning of the COVID-19 pandemic in Spain.	Sociodemographic variables, working conditions, training, availability of PPE, health effects, and pandemic evolution	None
Pinedo, R	To identify factors that influenced adults' mental health during the pandemic	Sociodemographic variables, household variables, daily planning and activities, confinement, number and reason of outings, healthy habits	MHI-5, CHS-5, SESLA-S
Planchuelo-Gómez, A	To analyse the longitudinal evolution of the levels of anxiety, depression, stress and the psychological impact of the COVID-19 crisis and confinement from the outbreak to the beginning of the relaxation of the lockdown measures in Spain.	Sociodemographic variables, personal situation during the confinement, psychological/psychiatric treatment, psychoactive medication, confinement impact on personal and social relationships, media consumption about COVID-19, trust in government and scientists, physical activity before and during confinement, perceived, COVID-19 infection and survival	DASS-21 and IES
Pouso, S	To test whether, during the first wave of the COVID-19 outbreak, people who maintained direct and/or indirect contact with outdoor spaces coped better with lockdown measures in terms of fewer symptoms of poor mental health and better maintenance of positive mood	Sociodemographic variables, general accessibility, Individual accessibility, perceptions related to views from home and to access to outdoor spaces	PHQ-4, Plutchik's wheel of emotions
Rodríguez-Barranco, M	To clarify the main causes of transmission of the COVID-19 disease during confinement and some risk factors, to detect the critical points of exposure to the virus	Sociodemographic variables, home characteristics, pets, work activity, protection, mobility, health conditions	None
Rodríguez-Larrad, A	To analyse the changes in patterns of physical activity and sedentary behaviours in Spanish university students before and during the confinement with special focus on gender.<br>To analyse the enjoyment, the tools used and motivation and impediments to do physical activity	Sociodemographic variables, physical activity and sedentary behaviours, academic and anthropometric data	IPAQ Short Form
Rodríguez-Pérez, C	To evaluate whether dietary behaviours of the Spanish adult population were changed during the COVID-19 outbreak confinement	Sociodemographic variables, consumption frequency of selected foods, general food, and habits	MEDAS
Rodriguez-Ruiz, E	To assess the impact of COVID-19 pandemic on MD among HCPs in Spanish ICUs.	Sociodemographic variables and work-related variables, work absence due to coronavirus infection or due to psychologic stress, anxiety, and/or depression	MMD-HP
Romero, CS	To assess the psychological impact of the COVID-19 pandemic in Spanish healthcare workers.	Sociodemographic variables and mental health variables	Psychological assessment tests, Healthcare Stressful Test, Coping Strategies Inventory, Font-Roja Questionnaire and Trait Meta-Mood Scale

**First author**	**Objective/s**	**Information blocks**	**Scales/composites variables (Abbreviations in Supplementary Table 2)**
Salas-Nicás, S	To describe the impact of COVID-19 on the working conditions and health of wage-earners in Spain.	Job, psychosocial, quality of life and alcohol and other drugs	ERP16, COPSOQ3, SF-36, EDADES
Sánchez-Cantalejo,C ; Cabrera-León, A	To determine the magnitude, characteristics, and evolution of the COVID-19 impact on overall health as well as socioeconomic, psychosocial, behavioural, occupational, environmental, and clinical determinants of both general and vulnerable population	Sociodemographic variables, household and housing variables, time use, cohabitation, health and emotional well-being, habits and lifestyle, and economic situation	Emotional well-being, social and emotional support, body mass index, social class
Valiente C	To evaluate the levels of anxiety, depression, and well-being in a Spanish sample during the confinement, and identify potential predictor variables associated to experiencing both clinical levels of distress and well-being	Sociodemographic variables and living conditions, anxiety and economic, threat related to COVID–19, health characteristics, increased substances-use scale, TILS	TILS
Vall-Roqué H	To determine the impact of COVID-19 lockdown on SNS use and to explore whether SNS use is associated with body image disturbances and low self-esteem	Sociodemographic variables, social network sites use, COVID-19 and lockdown-related information	Rosenberg self-esteem scale, Eating Disorder Inventory-3
Villanueva, VJ	To establish whether alcohol risk consumption varied during COVID-19 confinement and to establish how these variations manifested as a function of different sociodemographic variables	Sociodemographic variables	AUDIT-C, short version
Yélamos Agua, C	To identify the psychosocial impact of the pandemic-related lockdown on cancer patients, and thus make a first exploratory and empirical approximation to distress levels and related factors in such a vulnerable group	Sociodemographic variables, health and oncological condition, income, COVID-19 diagnosis or symptoms, attitude toward coronavirus, COVID-19 measures perception	Kessler K-6 scale

These variables and others not shown in this work can be consulted in the free access tool of Google Sheets [1].

[1] Disponible en: https://docs.google.com/spreadsheets/d/10hTOr1jC73tajIB-1jzHLalUfX8-2ZAl1GaApk6wRiI/edit?usp=sharing

Supplementary Table 2. Abbreviations used in Supplementary Table 1

Abbreviation	Meaning	Abbreviation	Meaning
AUDIT-C	Alcohol Use Disorders Identification Test	K10	Kessler Psychological Distress Scale
BMI	Body Mass Index	MEDAS	Mediterranean Diet Adherence Screener
CAGE-AID	CAGE Questionnaire Adapted to Include Drugs	MHI-5	Mental Health Inventory – 5
CES-D	Centre for Epidemiologic Studies Depression Scale	MMD-HP	Measure of Moral Distress for Healthcare Professionals
CHS-5	Coping Humour Scale	OSSS-3	Oslo Social Support Scale
CIDI	Composite International Diagnostic Interview	PASE	Physical Activity Scale for the Elderly
COPSOQ3	Copenhagen psychosocial questionnaire	PAVS	Physical Activity Vital Sign Scale
DASS-21	Depression, Anxiety and Stress Scales	PCL-5	Post-traumatic Stress Disorder Checklist
EDADES	Spanish survey on alcohol and other drugs	PHQ-8	Eight-item Patient Health Questionnaire depression scale
EEPAQ	Elderly EXERNET Physical Activity Questionnaire	PSQI	Pittsburgh Sleep Quality Index
EMAS	Multidimensional Scale of Perceived Social Support	PTSD	Posttraumatic Stress Disorder
EPAQ	EPIC-cohort questionnaire	RS-14	Resilience Scale
EQ-5D	European Quality of Life-5 Dimensions	SATLIFE	Satisfaction with life scale
EQLS	Eurofound's European Quality of Life Survey	SCS	Self-Compassion Scale
ERP16	Spanish psychosocial risks survey	SESLA-S	Social and Emotional Loneliness Scale for Adults-Short version
EU-SILC	EU Statistics on Income and Living Conditions	SF-36	Short Form-36 Health Survey
EuroQol-5D	European Quality of Life-5 Dimensions	SMILE-C	Short Multidimensional Inventory Lifestyle Evaluation
EWCS	European Working Conditions Survey	SRH	self-rated health
FACIT-Sp12	Functional assessment of chronic illness therapy—spiritual well-being scale	SWLS	Satisfaction with Life Scale
GAD-7	Generalized Anxiety Disorder scale	TILS	three-item Loneliness Scale
GHQ-12	12-Item General Health Questionnaire	UCLA-3	UCLA Loneliness Scale
HRQoL	Health-related quality of life	VAS	Visual Analog Scale
IES	Impact of Event Scale	WEMWBS	Warwick-Edinburgh Mental Well-being Scale
IES-R	Impact of Event Scale - Revised	WHO-5	The World Health Organization- Five Well-Being Index
InDI-D	Intersectional day-to-day discrimination Index	WHODAS	World Health Organization Disability Assessment Schedule
IPAQ	International Physical Activity Questionnaires	WMHICS	World Mental Health-International College Student
ISI	Insomnia Severity Index		
